# A New Class of Rhomboid Protease Inhibitors Discovered by Activity-Based Fluorescence Polarization

**DOI:** 10.1371/journal.pone.0072307

**Published:** 2013-08-22

**Authors:** Eliane V. Wolf, Annett Zeißler, Oliver Vosyka, Evelyn Zeiler, Stephan Sieber, Steven H. L. Verhelst

**Affiliations:** 1 Lehrstuhl für Chemie der Biopolymere, Technische Universität München, Freising, Germany; 2 Center for Integrated Protein Science Munich, Department Chemie, Institute of Advanced Studies, Technische Universiät München, Garching, Germany; Ben-Gurion University of the Negev, Israel

## Abstract

Rhomboids are intramembrane serine proteases that play diverse biological roles, including some that are of potential therapeutical relevance. Up to date, rhomboid inhibitor assays are based on protein substrate cleavage. Although rhomboids have an overlapping substrate specificity, substrates cannot be used universally. To overcome the need for substrates, we developed a screening assay using fluorescence polarization activity-based protein profiling (FluoPol ABPP) that is compatible with membrane proteases. With FluoPol ABPP, we identified new inhibitors for the *E. coli* rhomboid GlpG. Among these was a structural class that has not yet been reported as rhomboid inhibitors: β-lactones. They form covalent and irreversible complexes with the active site serine of GlpG. The presence of alkyne handles on the β-lactones also allowed activity-based labeling. Overall, these molecules represent a new scaffold for future inhibitor and activity-based probe development, whereas the assay will allow inhibitor screening of ill-characterized membrane proteases.

## Introduction

Proteases catalyze the hydrolysis of peptide bonds in proteins and are involved in digestive as well as regulatory processes. In the human genome, approximately 2% of the genes code for proteases. While most proteases are soluble, a small fraction is membrane-embedded [1]. These intramembrane proteases differ from soluble proteases in a variety of aspects: They are composed of a number of transmembrane domains (TMDs) which harbor the catalytic residues with their active sites buried several Å into the membrane. Their substrates are transmembrane proteins that reside inside the membrane in a dormant form. Upon cleavage, most substrates release a soluble part into the cytosol or extracellular space. It is therefore not surprising that intramembrane proteases are involved in various signaling pathways [1]–[3].

There are three families of intramembrane proteases, classified according to their catalytic mechanism: intramembrane metalloproteases (exemplified by site 2 protease), intramembrane aspartic proteases (such as presenilin), and intramembrane serine proteases. The latter belong to the family of rhomboid proteins, containing active intramembrane proteases and inactive homologs. Rhomboids are found in all kingdoms of life [4], [5], but are functionally diverse. They take part in various distinct cellular processes such as the EGFR-signaling pathway in the fruit fly *Drosophila melanogaster* [6], quorum sensing in the Gram negative bacterium *Providencia stuartii* [7] and host cell infection by apicomplexan parasites [8]–[10]. Structurally, rhomboids are the best characterized intramembrane proteases. Several different crystal forms of the *E. coli* rhomboid GlpG have provided insight into the mechanism of intramembrane proteolysis [11]–[14]. However, a detailed picture of the rhomboid-substrate interaction is not available. As an alternative, crystal structures of covalent inhibitors bound to GlpG have revealed which areas and residues may play a role in primed and non-primed site interaction, and oxyanion stabilization [15]–[19]. The availability of inhibitors is also important for future functional studies. Moreover, potent and selective inhibitors may serve as lead structures for future drug design. Up to date, rhomboid inhibitors have been reported based on three distinct scaffolds: 4-chloro-isocoumarins [6], [15], [18], fluorophosphonates [16], [17], [20], and N-sulfonylated beta-lactams [21]. However, these are not selective enough to inhibit only rhomboids within the entire proteome. In addition, these inhibitors are also not promiscuous enough to inhibit rhomboids from different organisms equally well [18]. Therefore, it is still of great interest to find new types of inhibitors. In order to facilitate this search, various screening methods have been employed so far. All of these have relied on monitoring the cleavage of a substrate through gel-based [22]–[24], FRET [21] or MALDI mass spectrometry techniques [18]. However, a limitation of these methods is the availability of a matching protein or polypeptide substrate. Rhomboids from one species may cleave substrates from another species, but this is not a general rule. We therefore reasoned that it would be beneficial to develop an inhibitor assay for rhomboid proteases that does not rely on a substrate at all.

A few years ago Cravatt and co-workers developed a high-throughput inhibitor screening method that uses fluorescent activity-based probes (ABPs) [25]. ABPs are small molecules that covalently bind to the active form of an enzyme, but not to an inactivated or zymogen form [26], [27]. ABPs generally consist of a tag, a spacer and an electrophilic group that traps an active site nucleophile. The binding event can be detected by a variety of techniques, such as gel-scanning, biotin blot or fluorescent microscopy, depending on the tagging moiety [28]. When appended to a fluorescent dye, the binding of an ABP can be detected by fluorescence polarization [25]. This so-called fluorescence polarization activity-based protein profiling (FluoPol ABPP) has been used in inhibitor high-throughput screens (HTS) for a variety of poorly characterized enzymes [25], [29], [30]. We here report the first FluoPol ABPP screen against a membrane enzyme: the *E. coli* rhomboid GlpG. Using this method, we have found a novel class of inhibitors for rhomboid proteases: β-lactones. These compounds represent new scaffolds for future rhomboid inhibitor and ABP development.

## Materials and Methods

### Rhomboid Expression and Purification

Rhomboid expression and purification were performed as described previously [18], with minor modifications: cells were lysed by sonication (5 min total time, 2 s pulse and 5 s pause alternating, 50% amplitude). Rhomboid protein concentration was determined by DC protein assay (Bio-Rad).

### Fluorescence Polarization Assay (FluoPol ABPP)

500 nM rhomboid in 99 µl of 50 mM HEPES (pH 7.3) containing 0.01% (w/v) Pluronic F-127 (Invitrogen) and 0.0125% (v/v) Triton X-100 were incubated with 100 µM of compound or DMSO for 30 min at 37°C shaking in a black 96-well plate. Then the FP-R probe (fluorophosphonate FP-rhodamine) was added to a final concentration of 75 nM and the measurement immediately started. The plates were read at 37°C in a Polarstar Omega Fluorescence Polarimeter (BMG Labtech) for up to 7 h in continuous intervals.

### Data Evaluation

Each sample was baseline corrected by subtraction of the starting value from the polarization value at 4 h. The polarization value of the GlpG S201A was then subtracted from all samples to obtain the assay-window. The value of GlpG WT was then defined as 100% value.

### Z’-determination

The Z’factor [31] was used to evaluate and validate the suitability of the rhomboid FluoPol assay for high-throughput screening using ten positive controls of the wild-type GlpG and ten negative controls of the inactive S201A mutant.

### Competitive Activity-based Probe Profiling

For the competitive activity-based probe profiling (competitive ABPP) experiment 45 nM of rhomboid in 20 µl of 50 mM HEPES (pH 7.4) containing 10% glycerol and 0.0125% DDM were incubated for 30 min at 37°C shaking with 100 µM of the compound or an equal volume of DMSO. Then either probe EK2 or FP-R was added to a final concentration of 1 µM and incubated for further 30 min at 37°C shaking in the dark. The labeling reaction was stopped with 4× SDS-sample buffer and 10 µl of the reaction (15 ng protein per lane) was applied to a 15% SDS-polyacrylamide gel and run for 1 h 10 min at 200V. The gel was visualized on a fluorescence scanner (Typhoon, Trio +).

### Substrate Cleavage

The TatA protein from *Providencia stuartii* was used for generation of a fluorescence substrate. The substrate was constructed to include a His6-tag for purification as well as a LPRTG-motif for sortase-mediated protein labeling. It was expressed and purified as described above. For the labeling reaction, 33 µM of the unlabeled substrate, 50 µM sortase A from *S. aureus* [32] and 500 µM of the label NH_2_-Ala-Ala-Ahx-Lys(TAMRA) [32] were added together in labeling buffer (50 mM Tris, pH 7.5 containing 150 mM NaCl, 10 mM CaCl_2_ and 0.05% (w/v) DDM) and allowed to react o/n at 37°C in the dark. During the labeling reaction the His6-tag of the substrate was exchanged for the TAMRA-label, so that labeled substrate did not contain a His6-tag. This allowed removal of sortase and unlabeled substrate by incubation of the labeling reaction with Ni-NTA agarose beads. The supernatant was applied to a ZEBA-spin desalting column (VWR; MW-cut-off 7 kDa) to remove excess label. 40 µL of a 500 nM rhomboid solution (in 50 mM HEPES (pH 7.4) containing 10% (v/v) glycerol and 0.0125% (w/v) DDM) were incubated for 30 min at 37°C shaking with 100 µM of the compounds or DMSO. Then fluorescently labeled rhomboid substrate TatA (*Providencia stuartii*) was added to a final concentration of 83 nM. The cleavage reaction was allowed to take place o/n at 37°C shaking in the dark and stopped by the addition of 4× SDS-sample buffer. 10 µl of each reaction (10 ng protein per lane) was applied to a 15% SDS-polyacrylamide gel and run for 1 h 10 min at 200V. The gel was visualized on a fluorescence scanner (Typhoon, Trio +).

### IC_50_ Determination

For the determination of the apparent IC_50_, 500 nM rhomboid in 99 µl of 50 mM HEPES (pH 7.4) containing 0.01% (w/v) Pluronic F-127 (Invitrogen) and 0.0125% (v/v) Triton X-100 were incubated with a range of concentrations of the compounds or an equal volume of DMSO for 30 min at 37°C shaking in a black 96-well plate. Then the FP-R probe was added to a final concentration of 75 nM and the measurement immediately started. The plates were read at 37°C in a Polarstar Omega Fluorescence Polarimeter (BMG LabTech) for up to 7 h in continuous intervals. The log(compound in nM) was plotted against the % remaining active enzyme.

### Reversibility Check

To assess the reversibility of the hit compounds, 500 nM rhomboid in 100 µl of 50 mM HEPES (pH 7.3) containing 10% (v/v) glycerol and 0.0125% (w/v) DDM were incubated for 30 min at 37°C shaking with 100 µM of the hit compound, the two false positive hits or an equal volume of DMSO. The sample was then applied to a ZEBA-spin column to remove unbound compound. The flow-through was then incubated with 1 µM of EK2 for 30 min at 37°C shaking in the dark. 20 µl of each sample were used for visualization on a fluorescence gel.

### Copper-mediated Click Reaction

For the two-step azide-alkyne cycloaddition, 36 nM of rhomboid in 50 µl of 50 mM phosphate buffer (pH 7.4) containing 0.0125% (w/v) DDM were incubated with 100 µM of **31** and **43** or an equal volume of DMSO for 30 min at 37°C. Then 0.5 µl each of 5 mM TAMRA-azide (in DMSO), 100 mM TCEP (in H_2_O), 100 mM CuSO_4_ (in H_2_O) and 1.7 mM TBTA (in DMSO) were added and the reaction incubated for 1 h at 37°C in the dark. The reaction was stopped by the addition of 4× SDS-sample buffer and 10 µl (12 ng of total protein) analyzed on a SDS-polyacrylamide gel.

## Results

### Development of a Fluorescence Polarization Assay for Rhomboids

**Figure 1:**
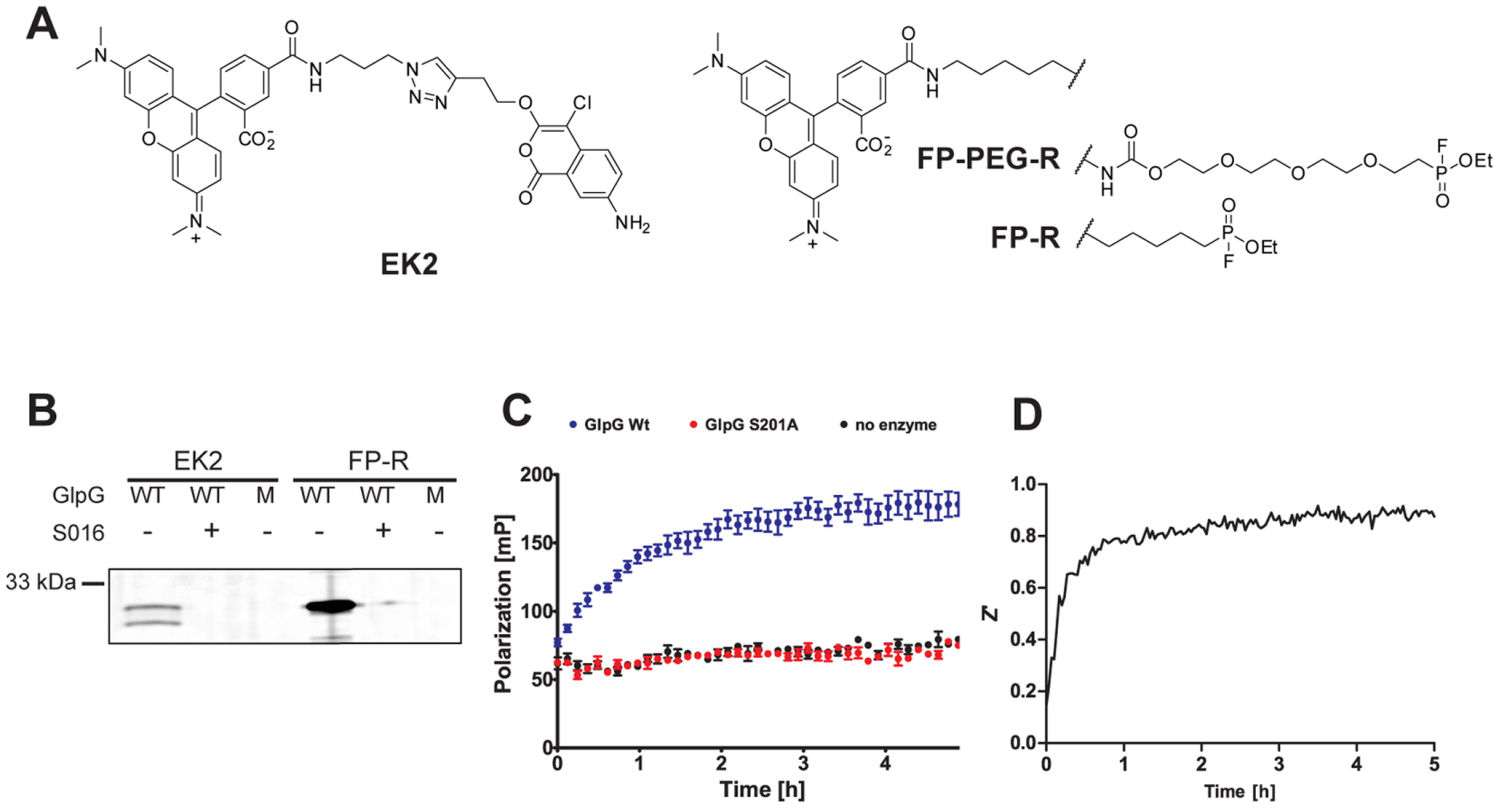
Development of a rhomboid FluoPol ABPP assay. (A) Chemical structures of the ABPs EK2 and FP-R/FP-PEG-R. (B) 45 nM wild-type (WT) GlpG or the inactive S201A mutant (M) were preincubated with 100 µM inhibitor S016 or 1% DMSO vehicle control (30 min) and subsequently incubated with 1 µM of probe EK2 or FP-R (30 min). (C) Fluorescent polarization measured over time using 75 nM FP-R and 500 nM GlpG WT, 500 nM S201A or plain buffer. The mean of quadruplicate measurements is depicted with standard error. (D) The development of the Z’ value over time during a 5 h run of rhomboid FluoPol ABPP.

**Figure 2:**
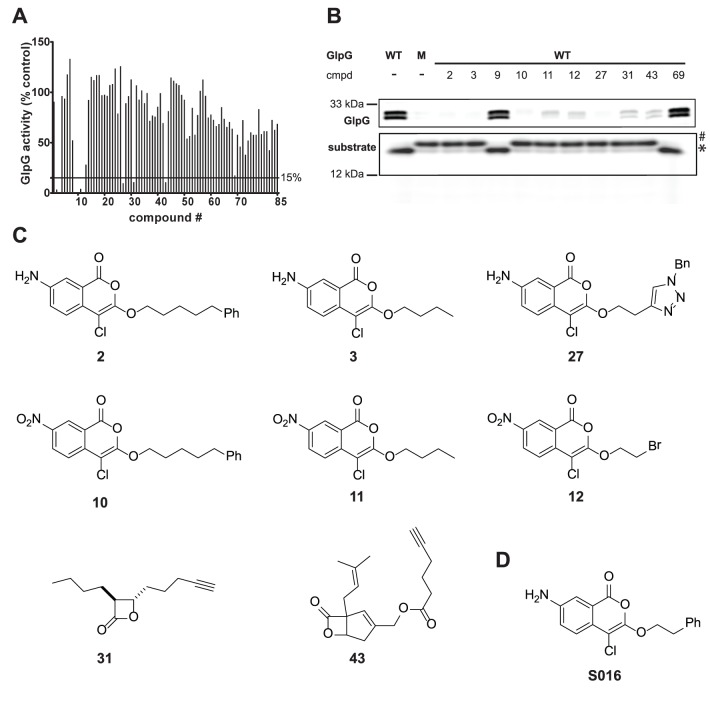
A small molecule screen by rhomboid FluoPol ABPP and subsequent hit confirmation. (A) Screening data of the 85 compounds investigated in the FluoPol assay. A 15% cut-off was set to select primary hits. For compounds **3**, **9**, **10**, **12** the slightly negative values are depicted as zero. (B) Confirmation of the potential inhibitors using GlpG wild-type (WT) or S201A mutant (M) by gel-based competitive ABPP using probe EK2 (upper panel) and by substrate cleavage. Uncleaved (#) and cleaved (*) substrate is indicated (lower panel). (C) Chemical structures of the verified hit compounds. (D) Chemical structure of the previously identified inhibitor S016.

Recently, we and others reported the first fluorescent ABPs for bacterial rhomboids [18], [20]. One ABP is the fluorophosphonate FP-PEG-rhodamine (FP-PEG-R; Figure 1A), the other one is based on the 4-chloro-isocoumarin scaffold (EK2; Figure 1A). Both FP-PEG-R and EK2 have only been used in gel-based applications. In view of previous work of the Cravatt laboratory [25], we expected that fluorescent rhomboid ABPs would be suitable for the development of a gel-free FluoPol ABPP screening method. Hence, we took EK2 and the commercially available fluorophosphonate FP-rhodamine (FP-R; Figure 1A) and verified whether these probes label rhomboid in an activity-based manner. Gratifyingly, both FP-R and EK2 labeled wild-type (WT) GlpG from *E. coli*, but not the inactive S201A mutant (Figure 1B). Labeling was also prevented by pre-inhibition of GlpG WT with the isocoumarin inhibitor S016, which we have identidfied in a previous MALDI-based screen (Figure 1B; see for structure Figure 2D) [18]. FP-R gave rise to a more intense labeling, probably due to the higher reactivity of the fluorophosphonate electrophile compared to the isocoumarin. We therefore chose this probe for subsequent FluoPol ABPP experiments. Until now FluoPol ABPP has only been performed on soluble enzymes without the presence of detergents. Hence, our initial experiments were focused on the optimization of FluoPol ABPP for usage with intramembrane proteases, which require the presence of detergents during their solubilization and purification. We found that detergents can interfere with FluoPol ABPP and lead to an unstable polarization signal over time (Figure S1). We tested a variety of conditions including different concentrations of dodecyl maltoside and Triton X-100 and found that a low amount of Triton X-100 (0.0125%) together with 0.01% Pluronic F127 gave robust and reproducible signals (Figure 1C). For assay quality assessment, we determined the Z’ value over time by measuring the fluorescence polarization signal for ten positive controls of the wild-type GlpG and ten negative controls of the inactive S201A mutant (Figure 1D). After 4 h, the Z’ reaches a value larger than 0.9, which is excellent for screening compound libraries [31].

### Screening of Small Molecules by Rhomboid FluoPol ABPP

Having determined the optimal assay conditions, we screened the *E. coli* rhomboid GlpG against a set of 85 small molecules. The molecules contained reactive electrophiles including isocoumarins [33], phosphonates, phosphoramidates, β-lactones [34], [35], β-sultams, epoxides and thiiranes [36] (Table S1). In duplicate screens, nine compounds gave 15% or less of the wild-type signal and were taken as primary hits together with an additional compound closely above the 15% mark (Figure 2A). Next, we conducted two different assays to confirm the hits. First, we performed in-gel competition experiments by pre-incubating GlpG with the small molecule compound and subsequent labeling with an ABP. To make sure that the hits are not dependent on the nature of the ABP we employed the isocoumarin EK2 as probe for the gel-based competitive ABPP (Figure 2B, upper panel). Second, to make sure that the rhomboid inhibition was not only an inhibition of labeling, but of the general proteolytic capacity of GlpG, we also tested cleavage of a fluorophore-tagged rhomboid substrate (Figure 2B, lower panel).

We found compounds **9** and **69**, which are two diphenyl phosphonates, to be false positives (Figure 2B). They did not block EK2 labeling and did not inhibit substrate cleavage. The other compounds –**2**, **3**, **10**, **11**, **12**, **27**, **31** and **43**– were confirmed as GlpG inhibitors: They were able to block ABP labeling as well as substrate cleavage. Their structures are shown in Figure 2C. Compounds **2**, **3**, **10**, **11**, **12** and **27** contain the 4-chloro-isocoumarin scaffold, which has been reported to yield rhomboid inhibitors before [6], [15], [18]. Interestingly, compound **31** is a monocyclic and compound **43** is a bicyclic β-lactone. The β-lactone scaffold represents a novel class of rhomboid inhibitors.

### Further Studies on the Hit Compounds

To further investigate the confirmed hit compounds, we determined the apparent IC_50_ by FluoPol ABPP (Table 1 and Figure S2). We included the isocoumarin S016 which we have characterized previously using a MALDI-based assay (Figure 2D) [18]. The apparent IC_50_ of S016 was 1.1 µM, which is in accordance with the value found before. Compounds **2**, **3** and **27** show a similar IC_50_, which is not surprising, since they have a close structural resemblance to S016. The potency of compounds **10**, **11** and **12** is approximately one order of magnitude lower. These isocoumarins have a nitro group instead of an amine, and confirm our previous finding that the amino group at the 7-position of the isocoumarin scaffold is important for potent and stable inhibition [18]. The β-lactones **31** and **43** have an apparent IC_50_ of 26 and 44 µM, respectively. Although weaker inhibitors than the 4-chloro-isocoumarins, future optimization by modifying the two substituents on the β-lactone scaffold, may lead to higher potency. To get a first idea for the specificity of the two β-lactones, we determined their IC_50_ against two canonical soluble serine proteases: bovine chymotrypsin and bovine trypsin. Against these targets, the two β-lactones showed an apparent IC_50_>50 µM against chymotrypsin and >150 µM against trypsin.

**Table 1:** Apparent IC_50_ (µM) of the hit compounds and S016, determined in duplicate measurements by FluoPol ABPP.

compound	apparent IC_50_
S016	1.1±0.56
2	0.75±0.21
3	0.40±0.10
10	5.2±0.80
11	5.5±0.55
12	8.4±1.7
27	3.1±0.91
31	26±5.8
43	44±10

**Figure 3:**
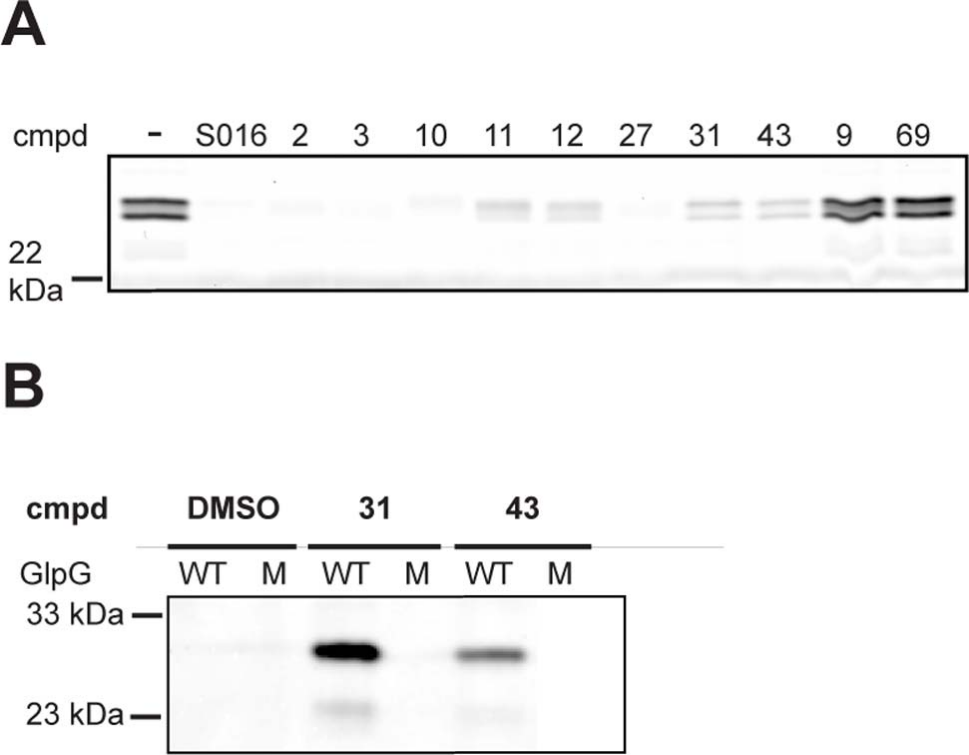
Determination of the inhibition mechanism of the hit compounds. (A) Reversibility study by incubation of 500 nM GlpG WT with 100 µM of hit compounds, the two false positives, S016 or 1% DMSO vehicle control, followed by gel filtration and subsequent labeling with 1 µM EK2. (B) Tandem labeling of GlpG by the two hit compounds **31** and 43∶36 nM of GlpG wild-type (WT) or S201A mutant (M) was incubated with 100 µM the hit compounds or 1% DMSO vehicle control, followed by copper-mediated click reaction to attach a TAMRA-azide.

Both 4-chloro-isocoumarins and β-lactones are electrophiles that can covalently and irreversibly react with active site serine residues of serine hydrolases [37]. To confirm whether the newly found 4-chloro-isocoumarins and β-lactones are indeed irreversible inhibitors, we pre-incubated GlpG with the hit compounds and the two false positives as negative controls, performed a gel filtration to remove non-covalently bound molecules and labeled the flow-through with the ABP EK2. As expected from their reported mechanism of action with soluble serine hydrolases, all verified hit compounds turned out to be irreversible inhibitors (Figure 3A).

### Usage of β-lactones as Activity-based Probes

The two β-lactones **31** and **43** both contain an alkyne handle, making them suitable for tandem, activity-based labeling. In tandem labeling, the enzyme is first reacted with the probe, which covalently binds to the active site. During a second step the covalent probe-enzyme complex is functionalized with a visualization tag, here through copper-catalyzed click chemistry with an azide derivative of a rhodamine fluorophore. Wild-type GlpG, but not the inactive S201A mutant was visualized as a fluorescent gel band by both β-lactones, confirming covalent and activity-dependent labeling. The β-lactones **31** and **43** therefore represent two new ABPs for the rhomboid GlpG (Figure 3B). Experiments on the application of these probes in membrane preparations and live cells are subject of future investigation.

## Discussion

In the last decade, small molecule ABPs have substantially impacted protease research, with applications ranging from activity profiling to target discovery and fluorescent imaging [38]. ABPs have also facilitated HTS for ill-characterized enzymes using fluorescent polarization [25]. This HTS has been executed on soluble, but not on membrane enzymes. Recent reports of the first ABPs for intramembrane proteases from the rhomboid family have therefore urged us to investigate FluoPol ABPP for use with membrane enzymes. We have managed this by employing a low concentration of a mild detergent and also found that the surfactant Pluronic F-127 is essential for a good signal-to-noise ratio, probably by facilitating the solubilization of the fluorescent dye. Overall, this resulted in an HTS compatible assay with a high Z’-value of 0.9. We are confident that the assay will enable the screening of other poorly characterized membrane-anchored or membrane embedded enzymes. The screening of rhomboids from different organisms is subject of our future research efforts.

The special advantage of FluoPol ABPP is that it does not require a substrate, but uses a broad-spectrum ABP. For rhomboids, no small molecule fluorogenic or chromogenic substrates are available as for soluble proteases. One FRET-based polypeptide has been used for screening, but this cannot be used universally. Protein substrates are still the standard assay technique to monitor rhomboid activity. However, the detection of cleavage of these substrates is laborious. Hence, the development and optimization of fluorescent ABPs for rhomboids and other membrane enzymes will likely assist inhibitor discovery for such enzymes.

Since the discovery of rhomboids as intramembrane proteases in 2001, inhibitor development has gained momentum slowly. Originally, only the broad spectrum inhibitor 3,4-dichloroisocoumarin (DCI) was found to inhibit rhomboids. Up to date, the known rhomboid inhibitors are based on three main scaffolds: 4-chloro-isocoumarins, *N*-sulfonylated-β-lactams and fluorophosphonates. Fluorophosphonates are highly reactive and non-selective reagents. FP-R, for example, reacts with 82% of all mouse metabolic serine hydrolases [39], which makes it an excellent broad-spectrum ABP. The rhomboid inhibitors based on 4-chloro-isocoumarins have gone through several optimization steps, from the weakly inhibiting DCI, to JLK-6 [15] and S016 [18], which is currently the most potent isocoumarin inhibitor for the *E. coli* rhomboid GlpG. Still, S016 is more potent against chymotrypsin than against GlpG [18]. The β-lactone scaffold that we have found here, is structurally related to β-lactams. β-lactones are more reactive than β-lactams, and unsurprisingly, β-lactams only act as rhomboid inhibitors when activated with a *N*-sulfonyl group [21]. The β-lactones **31** and **43** are less potent than the 4-chloro-isocoumarin S016, but they have a higher potency against GlpG than against trypsin and chymotrypsin. Hence, β-lactones may have the potential to be more selective inhibitors than 4-chloro-isocoumarins. Although compounds **31** and **43** also target other serine hydrolases [34], [35], the β-lactone scaffold can be readily influenced in its selectivity by changing the substituents on the lactone ring. Compound **43** for example, is an acylated form of **44**, the natural product vibralactone. Vibralactone is inactive against rhomboid, probably due to the presence of a polar hydroxyl group that may result in unfavourable interactions with the hydrophobic rhomboid TMDs. When this hydroxyl group is blocked as an ester function in compound **43**, it yields an active inhibitor. These structures illustrate the possibility to optimize the β-lactone scaffold for usage against rhomboids.

We have shown that the β-lactones covalently and irreversibly react with the active site serine of GlpG. This makes them well suitable for use as ‘warheads’ for ABPs. Compounds **31** and **43** contain an alkyne group in their structure, amenable to click chemistry-mediated derivatization. This feature allowed the direct on-gel visualization of the active rhomboid form. Hence, this study adds two new ABPs to the rhomboid chemical toolbox. Since β-lactones have already been successfully used for ABPP of serine hydrolases in lysates and live bacterial cells [40], [41], we expect them to be useful tools for the *in vivo* functional study of bacterial rhomboids.

## Supporting Information

Figure S1 **FluoPol ABPP under different conditions.** The influence of *n*-dodecyl β-D-maltoside (DDM) on the FluoPol ABPP assay quality, tested with 500 nM GlpG and 75 nM FP-R over 1 h.
(TIF)

Figure S2 **Apparent IC_50_-curves for the hit compounds measured by FluoPol ABPP.**
(TIF)

Table S1 **List of compounds used in the Rhomboid FluoPol ABPP Screen.**
(DOCX)
