# Supplementary figures and images for: A New Class of Rhomboid Protease Inhibitors Discovered by Activity-Based Fluorescence Polarization
Source: PLoS One. 2013 Aug 22;8(8):e72307. doi: 10.1371/journal.pone.0072307 (PMC3750051; doi:10.1371/journal.pone.0072307)

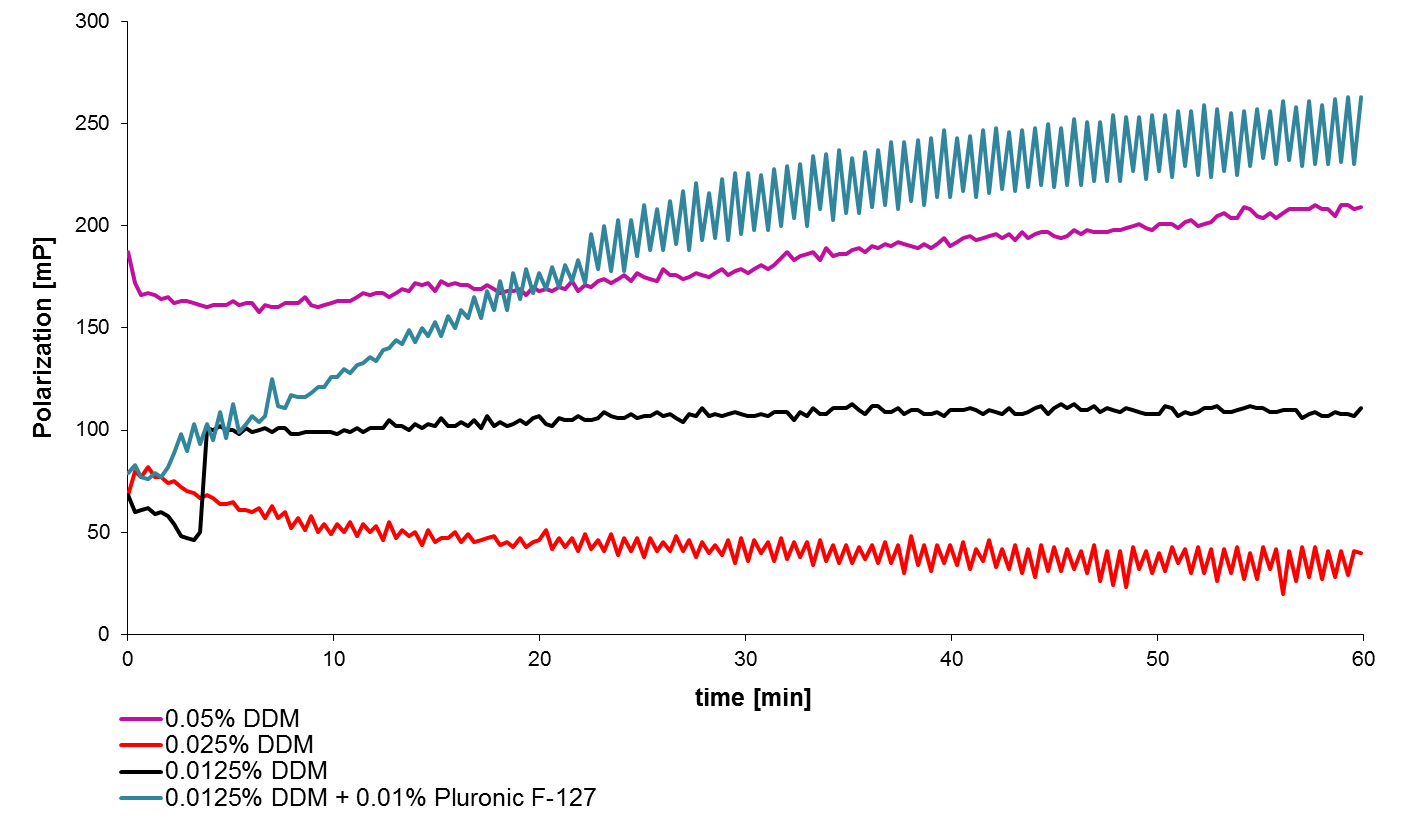

Supplement: Figure S1 — FluoPol ABPP under different conditions. The influence of n-dodecyl β-D-maltoside (DDM) on the FluoPol ABPP assay quality, tested with 500 nM GlpG and 75 nM FP-R over 1 h. (TIF) [file pone.0072307.s001.tif]

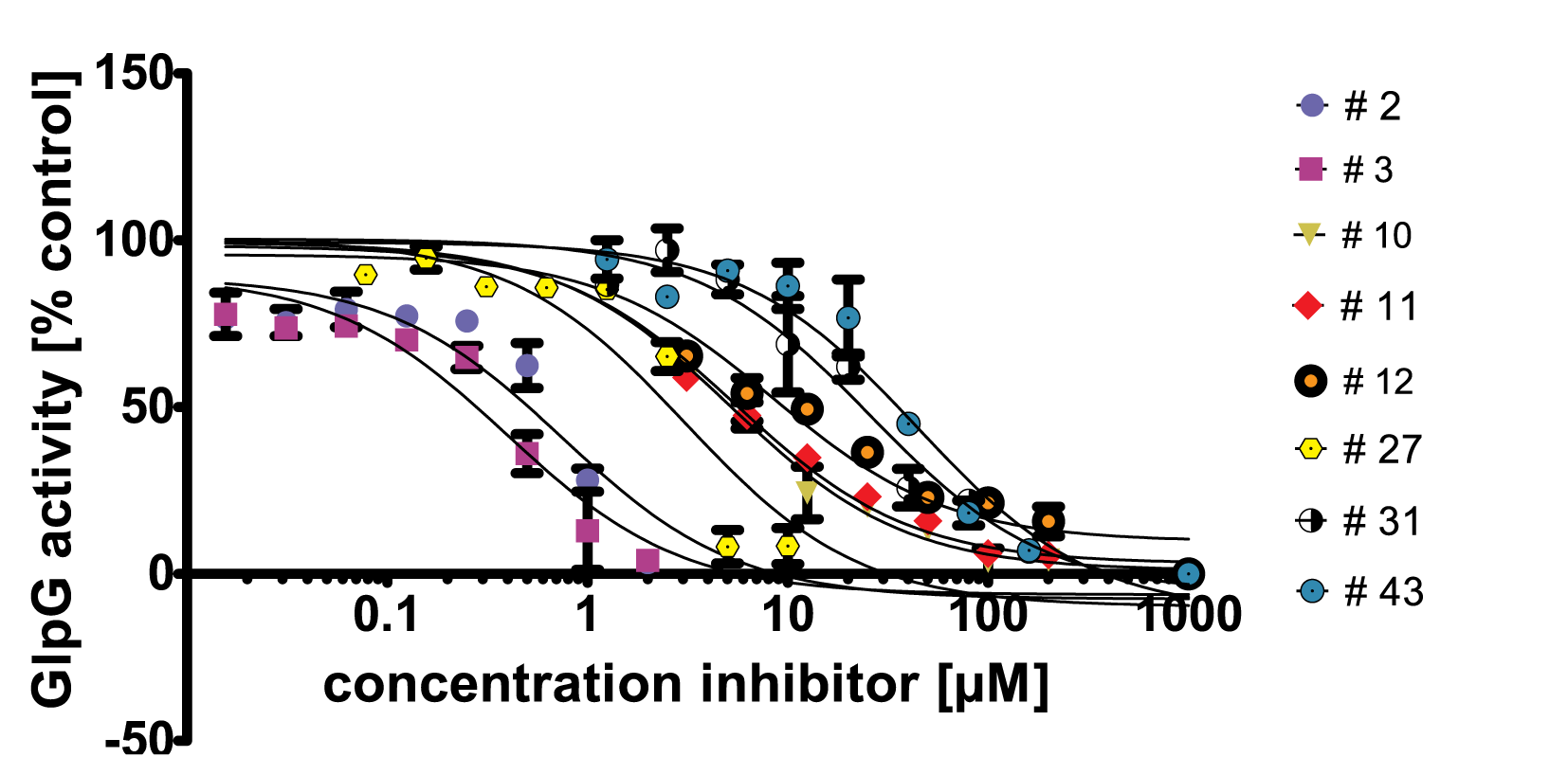

Supplement: Figure S2 — Apparent IC50-curves for the hit compounds measured by FluoPol ABPP. (TIF) [file pone.0072307.s002.tif]
