# Supplementary material for: A New Class of Rhomboid Protease Inhibitors Discovered by Activity-Based Fluorescence Polarization
Source: PLoS One. 2013 Aug 22;8(8):e72307. doi: 10.1371/journal.pone.0072307 (PMC3750051; doi:10.1371/journal.pone.0072307)
Supplement: Table S1 — List of compounds used in the Rhomboid FluoPol ABPP Screen. (DOCX) [file pone.0072307.s003.docx]

SUPPLEMENTARY TABLES

**Table S1. List of compounds used in the Rhomboid FluoPol ABPP Screen.**

| **#** | **structure** |
| --- | --- |
| **1** |  |
| **2** |  |
| **3** |  |
| **4** |  |
| **5** |  |
| **6** |  |
| **7** |  |
| **8** |  |
| **9** |  |
| **10** |  |
| **11** |  |
| **12** |  |
| **13** |  |
| **14** |  |
| **15** |  |
| **16** |  |
| **17** |  |
| **18** |  |
| **19** |  |
| **20** |  |
| **21** |  |
| **22** |  |
| **23** |  |
| **24** |  |
| **25** |  |
| **26** |  |
| **27** |  |
| **28** |  |
| **29** |  |
| **30** |  |
| **31** |  |
| **32** |  |
| **33** |  |
| **34** |  |
| **35** |  |
| **36** |  |
| **37** |  |
| **38** |  |
| **39** |  |
| **40** |  |
| **41** |  |
| **42** |  |
| **43** |  |
| **44** |  |
| **45** |  |
| **46** |  |
| **47** |  |
| **48** |  |
| **49** |  |
| **50** |  |
| **51** |  |
| **52** |  |
| **53** |  |
| **54** |  |
| **55** |  |
| **56** |  |
| **57** |  |
| **58** |  |
| **59** |  |
| **60** |  |
| **61** |  |
| **62** |  |
| **63** |  |
| **64** |  |
| **65** |  |
| **66** |  |
| **67** |  |
| **68** |  |
| **69** |  |
| **70** |  |
| **71** |  |
| **72** |  |
| **73** |  |
| **74** |  |
| **75** |  |
| **76** |  |
| **77** |  |
| **78** |  |
| **79** |  |
| **80** |  |
| **81** |  |
| **82** |  |
| **83** |  |
| **84** |  |
| **85** |  |
